# Identification of Intracellular β-Barrel Residues Involved in Ion Selectivity in the Mechanosensitive Channel of *Thermoanaerobacter tengcongensis*

**DOI:** 10.3389/fphys.2017.00832

**Published:** 2017-10-25

**Authors:** Yingcai Song, Bing Zhang, Fei Guo, Maojun Yang, Yang Li, Zhi-Qiang Liu

**Affiliations:** ^1^Department of Anaesthesiology, Shanghai First Maternity and Infant Hospital, Tongji University School of Medicine, Shanghai, China; ^2^Key Laboratory of Receptor Research, Shanghai Institute of Materia Medica, Chinese Academy of Sciences, Shanghai, China; ^3^Key Laboratory for Protein Sciences of Ministry of Education, Tsinghua-Peking Center for Life Sciences, School of Life Sciences, Tsinghua University, Beijing, China

**Keywords:** *Tt*MscS, anion selectivity, β-barrel, selective filter, patch-clamp, single mutation

## Abstract

The mechanosensitive channel of small conductance (MscS) is a bacterial membrane pore that senses membrane tension and protects cells from lysis by releasing osmolytes. MscS is a homoheptameric channel with a cytoplasmic domain with seven portals and a β-barrel opening to the cytoplasm. *Tt*MscS, an MscS channel from *Thermoanaerobacter tengcongensis*, is an anion-selective channel. A previous study from our laboratory has defined the crucial role of β-barrel in the anion selectivity of *Tt*MscS (Zhang et al., 2012). However, the mechanistic details by which the β-barrel determines anion selectivity remain unclear. Here, using mutagenesis and patch-clamp recordings, we investigated the function and structural correlations between β-barrels and the anion selectivity of *Tt*MscS at the atomic level. Our results indicated that mutation of V274, a residue at the center of the inner wall of the β-barrel in *Tt*MscS, caused the anion selectivity of *Tt*MscS reverse to cation selectivity. Moreover, the electrostatic potential (T272) and physical size (L276) of residues in the inner wall of β-barrel also determine the anion selectivity of *Tt*MscS. In summary, the present study confirmed that the β-barrel region of *Tt*MscS acts as a “selective filter” that renders *Tt*MscS anion selectivity.

## Introduction

Mechanosensitive (MS) channels are membrane pores that are universally present in prokaryotes and eukaryotes (Martinac et al., 2008; Kung et al., 2010). These channels sense membrane tension and consequently accelerate the passage of osmolytes from areas of high concentration to those of low concentration (Kung, 2005). In bacteria, MS channels can be activated by turgor pressure. Like an “emergency valve,” when bacteria experience hypoosmotic shock, such as during rain, the channels open and release osmolytes along with water, thus alleviating cell turgor and protecting cells from lysis (Martinac et al., 1987). Additionally, a recent study has suggested that MS channels play a crucial role in the calcium regulation of bacteria with life-threatening hypoosmotic conditions (Cox et al., 2013), thereby ensuring bacterial survival across a wide range of external osmolarity levels. In mammals, MS channels have been implicated in numerous physiological processes, including touch, pain sensation, hearing, blood pressure control, micturition, tissue growth, cell volume regulation, and turgor control (Arnadóttir and Chalfie, 2010; Ranade et al., 2015).

The mechanosensitive channel of small conductance (MscS) is a homoheptameric channel that belongs to a family of pressure-sensitive channels. MscS channels are small conductance channels that open at a slight pressure of tens of mmHg; this pressure may result from an osmotic difference of only a few milliosmoles through the membrane (Martinac et al., 1987; Kung et al., 2010). This characteristic allows bacteria to detect circumstantial osmotic changes more efficiency.

Two crystal structures of *Ec*MscS (the MscS channel from *Escherichia coli*) have been solved. One is a wild-type *Ec*MscS structure that presumably represents a closed state of MscS (Bass et al., 2002), and the other is *Ec*MscS with an A106V mutation. This mutant has a substantial rotational rearrangement of the transmembrane (TM) region and an increased pore size; it is considered to reflect an open state of MscS (Wang et al., 2008). The MscS channels include two functional domains: a TM domain and a cytoplasmic domain. The TM domain enables the sensors to sense the tension exerted on the membrane or depolarization of the membrane potential (Bass et al., 2002; Bezanilla and Perozo, 2002). The cytoplasmic domain, with seven portals and a β-barrel (a distal pore at the bottom of MscS) opening to the cytoplasm, is conserved in the MscS family (Perozo and Rees, 2003; Schumann et al., 2004). However, the function of the cytoplasmic domain is unclear. Recent biophysical studies have suggested that the cytoplasmic vestibule undergoes significant conformational changes when this channel opens (Miller et al., 2003). In addition, the cytoplasmic domain has been reported to be involved in the adaptation and ion-selective process of the channel (Sotomayor et al., 2007; Gamini et al., 2011; Cox et al., 2013).

The crystal structure and functions of *Tt*MscS, an MscS channel from *Thermoanaerobacter tengcongensis* (*T. tengc*), has recently been reported by our team (Zhang et al., 2012). Although, *Tt*MscS has only 27% similarity to *Ec*MscS, their overall structural folds are similar. The entire structure of *Tt*MscS closely resembles the *Ec*MscS structure of the closed state except for two distinct cytoplasmic regions: the side portals and the bottom β-barrel. Sequence alignment combined with structural analysis revealed that three bulky residues, Phe157, Met243, and Trp246, lead to a much smaller portal for *Tt*MscS than *Ec*MscS. In contrast, the β-barrel in *Tt*MscS is much larger than that in *Ec*MscS. In addition, the pattern of electrostatic surface potentials inside the β-barrel pore and around the β-barrel region between *Tt*MscS and *Ec*MscS are opposite. The larger pore size and distinctive surface potential distribution indicate that the β-barrel is a dominant pathway for ion entry in *Tt*MscS (Zhang et al., 2012). However, the mechanistic details of the β-barrel structure to anion selection of *Tt*MscS are still unclear.

In this study, on the basis of structural analysis, we introduced several mutations at key residues in the inner wall of the β-barrel pore, aiming to change the physical size and electrostatic potential of the side chains of these key residues. Our electrophysiological results showed that key point mutations in the β-barrel attenuated or reversed the anion selectivity of *Tt*MscS. In conclusion, the present study defines several residues that are crucial for the anion selectivity of *Tt*MscS and supports the hypothesis that the β-barrel of *Tt*MscS acts as a “selective filter” that confers *Tt*MscS anion selectivity.

## Materials and methods

### Protein expression and purification

The gene encoding the MscS channel from *T. tengcongensis* (*Tt*MscS) was cloned into pET-21, a vector with an N-terminal 6 × His tag followed by a tobacco etch virus (TEV) protease cleavage site. The channel was expressed in BL21 cell cultures by induction with 0.4 mM isopropyl-β-d-thiogalactopyranoside (IPTG) at A_600_ ~ 1.0. The cells were cultured at 23°C for 18 h and collected. The collected bacteria were homogenized in lysis buffer (containing: 50 mM Tris · HCl and 200 mM NaCl; PH: 8.0) and lysed by sonication. The suspension was centrifuged (15,000 × g) for 15 min to remove cell debris. The supernatant was collected and centrifuged by ultracentrifugation (100,000 × g) for 1 h to obtain the membrane fraction (sediment). The membrane fraction was suspended in buffer A (containing 25 mM Tris · HCl, 20 mM imidazole and 500 mM NaCl; PH: 8.0), and Triton X-100 (0.5%, vol/vol) and PMSF (1 mM) were then added. The suspension was incubated at 4°C with slow stirring overnight. Then, the sample was subjected to ultracentrifugation (100,000 × g) for 30 min. The supernatant was harvested and loaded onto a Ni-NTA column (GE Healthcare) pre-equilibrated with buffer A containing 0.1% Triton X-100. Next, the column was washed with buffer A by adding 20 mM imidazole and 0.02% n-dodecyl-β-D-maltopyranoside (DDM) to detach any non-specific binding. The channel protein was washed with buffer B (containing 25 mM Tris·HCl, 300 mM imidazole, 500 mM NaCl, and 0.02% DDM, PH: 8.0), and the N-terminal 6 × His-tag was removed by TEV protease. The channel protein was concentrated and further purified by gel filtration (Superdex-200, GE Healthcare) with elution buffer (25 mM Tris·HCl, 200 mM NaCl, 5 mM DTT and 0.02% DDM; PH: 8.0). To confirm the purity of the protein, the peak fraction was collected and separated by SDS-PAGE and stained with Coomassie blue. The purified proteins were stored at 4°C for no more than 1 week for further functional studies.

### Preparation of giant liposomes and electrical recording

All lipids used in this study were obtained from Avanti Polar Lipids. The wild-type *Tt*MscS channel and mutant proteins were reconstituted into lipid vesicles composed of 1-pal-mitoyl-2-oleoyl-phosphatidylethanolamine (POPE, 7.5 mg/mL) and 1-palmitoyl-2-oleoyl-phosphatidylglycerol (POPG, 2.5 mg/mL) by using a previously described method (Li et al., 2007). To ensure single channel recording, the protein concentrations of lipids were all 150 μg/ml. Before electrophysiological analysis, the lipids were subjected to regular dehydrate and hydrate processes to obtain giant liposomes. We performed patch-clamp recording in asymmetrical solution with conditioning in 150 mM KCl, 500 mM sucrose, 5 mM K-HEPES (pH 7.0) in the bath solution, and 15 mM KCl, 500 mM sucrose, 5 mM K-HEPES (pH 7.0) in the pipette solution (the 500 sucrose provides osmotic protection, prevents giant liposomes from bursting and stabilizes the gigaohm seal formation). When measuring the preference for other anions, KCl was exchanged to the corresponding saline solution (KNO_3_, KF, KBr). Membrane voltages were controlled. After gigaohm seal (with a constant resistance of 3–8 GΩ) formation, the current was recorded with an Axopatch 200B amplifier with a Digidata 1322A analog-to-digital converter (Axon Instruments). Single channel currents were recorded in Clampex 10.2 software, filtered at 2 kHz, and digitized at 5 kHz. The data were analyzed in Clampfit 10.2 software, and the current amplitudes were measured by the difference between the cursor aligned at the baseline and peak of the current traces. The mechanical pressure was measured with a pressure monitor (PM015D, WPI).

### Estimate of the ion conductance and ion permeability ratio of *Tt*MscS channel

We plotted single-channel currents against voltage to acquire I–V curves. Ion conductance was estimated by the slope of the I–V curves. The permeability ratios were calculated using the following versions of the Goldman–Hodgkin–Katz (GHK) equation:

PX/PK=[K+]0-[K+]iexp(-ERevFRT)[X-]0exp(-ERevFRT)-[X-]i

where X is a monovalent anion. [X]_o_/[K^+^]_o_ and [X]_i_/[K^+^]_i_ are ion concentration on extracellular (cis-side) and intracellular sides (trans-side), respectively.

### Estimate of the free energy of activation of the *Tt*MscS channel

We estimated the free energy of activation of the *Tt*MscS channel protein by using a previously described method (Kloda and Martinac, 2001). Briefly, the open probability of the *Tt*MscS channel plotted against negative pressure was fitted to a Boltzmann distribution function given by:

$$\begin{array}{l} NP_{o}=NP_{omax}{\left[1+exp\;\alpha \;\left(p_{1/2}-p\right)\right]}^{-1} \end{array}$$

where *N* is the number of channels in the patch, and α is the sensitivity constant to negative pressure; *p* is the negative pressure applied to the pipette, and *p*_1/2_ is the negative pressure applied when the open probability of the channel is 0.5 (*P*_*o*_ = 0.5). *P*_*o*_ and *P*_*o*__*max*_ represent the open probability and maximum open probability of the channel, respectively.

A two-state Boltzmann model was used to fit the gating of MS channels, with the change of area *t*Δ*A* as the dominant energy term (Sukharev et al., 1999). The free energy Δ*G* is a linear function of membrane tension, as shown in the following equation:

$$\begin{array}{l} \Delta G=t\Delta A-\Delta G_{o}. \end{array}$$

where Δ*Go* is the difference in free energy between the closed and opened conformations of MS channels with the absence of the externally membrane tension. Δ*A* is the difference in membrane area occupied by open and closed channels at a given membrane tension, and *t*Δ*A* is the work for the gating of MS channels gated at the open probability of 0 < *P*_*o*_ < 1.

Because the Boltzmann function for the open probability of MS channels can be written as

$$\begin{array}{l} P_{o}/\left(1-P_{o}\right)=exp\;\left[\alpha \left(p-p_{1/2}\right)\right]=exp\;\left[\left(t\Delta A-\Delta G_{o}\right)/kT\right]. \end{array}$$

By using Laplace's law

$$\begin{array}{l} t-t_{1/2}=\left(p-p_{1/2}\right)\;\left(r/2\right). \end{array}$$

where *r* is the radius of curvature of the liposome patch shaped by the negative pressure applied to the pipette. Thus, when the open probability P_o_ = 0.5 (i.e., *p* = *p*_1/2_ and *t* = *t*_1/2_) the free energy difference Δ*G*_*o*_ = 0. Consequently, *t*_1/2_ = Δ*Go/*Δ*A* and *p*_1/2_ = *2*Δ*G*_*o*_*/r*Δ*A*, whereas α = *r*Δ*A/2kT*. The expression of the free energy for the activation of *Tt*MscS channel in this study is given by

$$\begin{array}{l} \Gamma_{MSC}=\alpha \;P_{1/2}=\Delta G_{o}/kT. \end{array}$$

### Statistical analyses

All data presented in this paper were expressed as the mean ± SEM. The ion selectivity (P_Cl_/P_K_) was estimated by using the Goldman–Hodgkin–Katz (GHK) equation. The P_X_/P_K_ ratios of *Tt*MscS in different anion solutions (**Figures 2C**, **5C**) were analyzed by using unpaired *t*-tests. The conductance (**Figure 2D**) and P_Cl_/P_K_ ratios of mutations (**Figures 4D**, **6B,D**) were analyzed using one-way ANOVA, and *post-hoc* LSD tests were performed.

## Results

### *Tt*MscS is a mechano-sensitive and anion-selective channel

**Figure 1:**
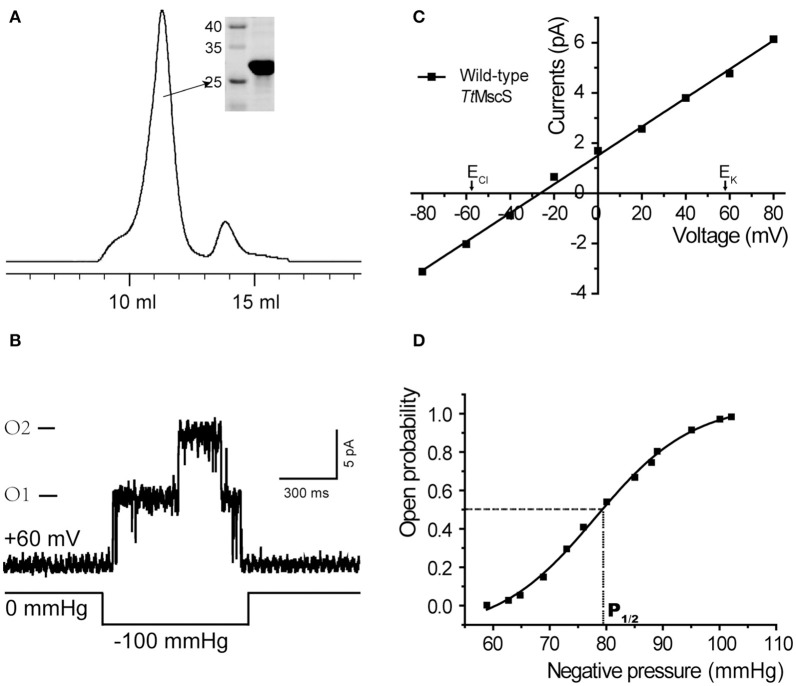
*Tt*MscS channels display strong mechano-sensitivity and anion selectivity. **(A)** Representative trace of *Tt*MscS in gel filtration chromatography: volume to peak was 11–12 ml; purity of protein was identified by SDS-PAGE gel was stained with Coomassie blue. **(B)** Single-channel traces of *Tt*MscS were recorded in asymmetric KCl solutions (bath/pipette: 150/15 mM) with a holding voltage of +60 mV; single or dual channels opened under negative pressure applied to the patch pipette. **(C)** I–V curve for wild-type *Tt*MscS recorded in asymmetric KCl solutions (bath/pipette: 150/15 mM). According to the GHK equation, the reversal potential of an ideal anion- or cation-selective channel was −58 or +58 mV. The reversal potential for *Tt*MscS was −26.7 ± 0.9 mV (mean ± SEM, *n* = 7). **(D)** Open probability of *Tt*MscS vs. negative pressure applied to the patch pipette fitted by the Boltzmann distribution function. P_1/2_ represents negative pressure at the point at which the open probability is 0.5.

We cloned the *Tt*MscS gene into the pET-21a vector. The *Tt*MscS channel was overexpressed in BL21 competent cells. Through gel filtration chromatography, we acquired high-purity *Tt*MscS protein (32 kDa) (Figure 1A). To detect the activity and physiological functions of *Tt*MscS, we reconstituted high-purity protein into lipid vesicles (giant liposome) and tested the electrophysiological properties by using a patch-clamp recording system. In patch-clamp recording experiments, the *Tt*MscS channel opened under negative pressure, thus reflecting a perfect mechanosensitive property (Figure 1B). The open probability plotted against negative pipette pressure was fitted to a Boltzmann distribution function (Figure 1D). The sensitivity parameter (1/α) of *Tt*MscS to pressure was 4.8 ± 0.9 mmHg, and P_1/2_, the pressure at the point when the channel open probability was 0.5, was 79.7 ± 5.2 mmHg (mean ± SEM, *n* = 3). Using Γ_*MSC*_ = α *P*_1/2_ = Δ*G*_*o*_/*kT*, we obtained the free energy of the channel activation ΔG_o_ = 17.5 ± 2.9 kT (see section Materials and Methods). The ion selectivity was measured with a reversal potential under asymmetric KCl solutions (15 mM KCl in pipette and 150 mM in the bath). *Tt*MscS exhibited an average reversal potential of −26.7 ± 0.9 mV (Figure 1C, mean ± SEM, *n* = 7). According to the GHK equation, we calculated the permeability ratio of *Tt*MscS for chloride vs. potassium P_Cl_/P_K_ ~ 4:1. The results suggested that *Tt*MscS is a mechano-sensitive, anion-selective ion channel.

### *Tt*MscS showed different degrees of preference for different anions

**Figure 2:**
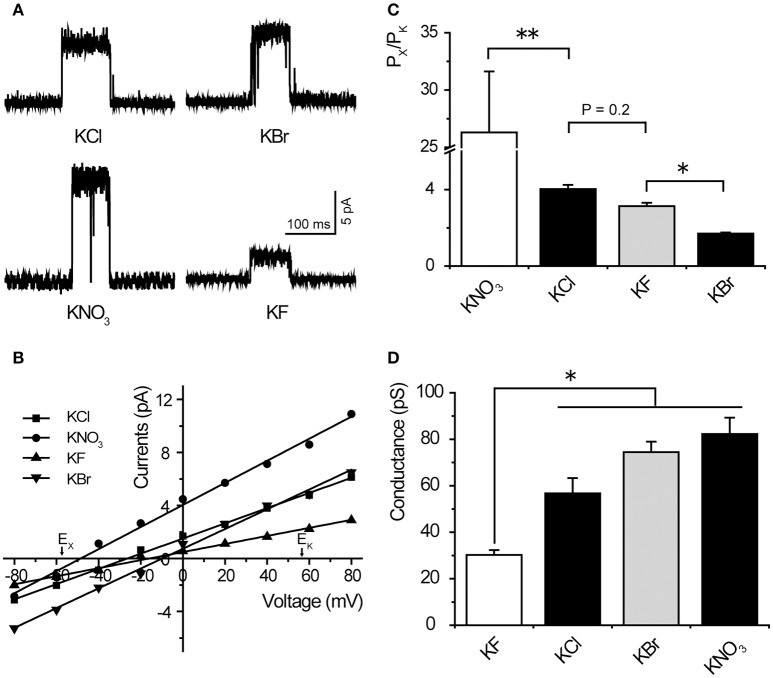
*Tt*MscS showed different preferences for different anions. **(A)** Single-channel traces of *Tt*MscS in different anion solutions (KNO_3_, KCl, KF, KBr) at +60 mV. **(B)** I–V curves for *Tt*MscS recorded in asymmetric anion solutions (KNO_3_, KCl, KF, KBr) (bath/pipette: 150/15 mM). The reversal potentials were −48.6 ± 1.9 mV (mean ± SEM, *n* = 5), −21.8 ± 1.5 mV (mean ± SEM, *n* = 5), and −11.8 ± 1.0 mV (mean ± SEM, *n* = 5) for KNO_3_, KF and KBr, respectively. **(C)** Ion selectivity (P_Cl_/P_K_) of *Tt*MscS in different asymmetric anion solutions (KNO_3_, KCl, KF, KBr). **(D)** Ion conductance of *Tt*MscS in different asymmetric anion solutions (KNO_3_, KCl, KF, KBr). ^*^p < 0.05, ^**^p < 0.01.

We demonstrated that *Tt*MscS showed a preference for Cl^−^ over K^+^. Therefore, we further investigated the preference of *Tt*MscS for other anions. To determine the anion preference of *Tt*MscS for various anions (${\text{NO}}_{3}^{-}$, F^−^, Br^−^), we exchanged other anions (${\text{NO}}_{3}^{-}$, F^−^, Br^−^) in asymmetric solutions and contrasted the differences of corresponding reversal potentials. Our results demonstrated that *Tt*MscS showed selective preferences for various anions, namely, ${\text{NO}}_{3}^{-}$ > Cl^−^ ≈ F^−^ > Br^−^ [Figures 2B, C, Table 1, unpaired *t*-test, *t*_(1, 10)_ = 5.047, *p* = 0.001 for KNO_3_ vs. KCl comparison; *t*_(1, 10)_ = 2.906, *p* = 0.2 for KCl vs. KF comparison; *t*_(1, 7)_ = 13.533, *p* = 0.033 for KF vs. KBr comparison]. Notably, *Tt*MscS showed much stronger selectivity for ${\text{NO}}_{3}^{-}$ (E_rev_ = −48.6 ± 1.9 mV, P_Cl_/P_K_ ~ 26:1, *n* = 5) than other anions, very similarly to the anion preference of the GABA receptor (P_Na_/P_K_/P_Cl_ ~ 0:0.03:1; Wotring et al., 2003).

**Table 1:** Summary of properties of wild-type *Tt*MscS in different anionic solutions.

	**E_rev_ (mV)**	**P_X_/P_K_**	**Conductance (γ, pS)**	***n***
KNO_3_	−48.6 ± 1.9	26.3 ± 5.3	82.3 ± 7.1	5
KCl	−26.7 ± 0.9	4.0 ± 0.2	56.8 ± 6.6	7
KF	−21.8 ± 0.5	3.0 ± 0.1	30.2 ± 2.1	5
KBr	−11.8 ± 1.0	1.7 ± 0.1	74.5 ± 4.5	4

Ion conductance is a basic property of ion channels (Bezanilla, 2008). We compared the ion conductance of *Tt*MscS performed with various anions (${\text{NO}}_{3}^{-}$, Cl^−^, F^−^, Br^−^). Unexpectedly, *Tt*MscS expressed much lower conductance to F^−^ than other anions (including Cl^−^, Br^−^, and ${\text{NO}}_{3}^{-}$) that are larger than F^−^ [Figures 2A,D, Table 1, one-way ANOVA, *F*_(3, 17)_ = 14.069, *p* < 0.001 for Figure 2D]. This result suggested that the conductance of ion channels is determined not only by the physical size of the ions but also by the interaction between ions and ion channels. The lowest conductance for F^−^ may be due to the large binding energy of F^−^ with the *Tt*MscS channel.

### β-Barrel region may confers the anion selectivity of *Tt*MscS

**Figure 3:**
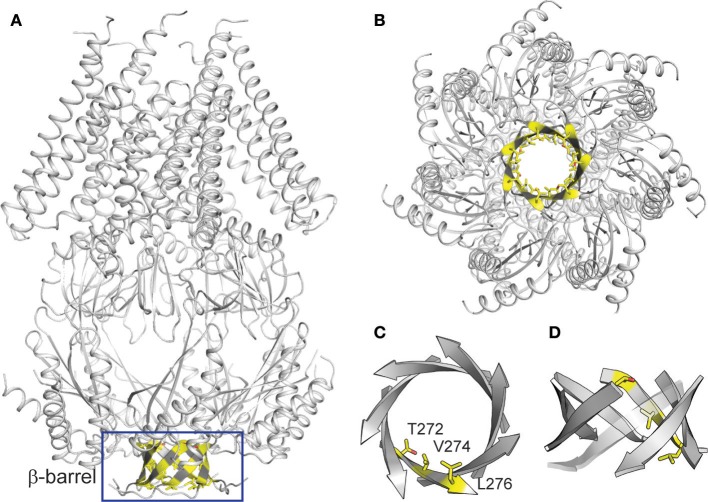
Structural analysis of *Tt*MscS**. (A)** overall structure of *Tt*MscS homoheptamer with a β-barrel at the distal end of *Tt*MscS. **(B)** Crystal structure of *Tt*MscS viewed from the bottom, with residues T272, V274, and L276 in the inner wall of β-barrel shown as sticks. **(C,D)** Amplified structure of the β-barrel viewed from the bottom **(C)** and cytoplasmic sides **(D)**. Three residues (T272, V274, L276) in one protomer are shown as sticks.

*Tt*MscS is a homoheptameric complex, each monomer of which consists of 286 amino acids. Like other MscS channels, the *Tt*MscS channel has two characteristic domains: a transmembrane domain and a cytoplasmic domain. The cytoplasmic domain comprises two distinct openings: the portals and the β-barrel (Figures 3A,B). Despite the similar overall structural fold between *Tt*MscS and *Ec*MscS, the portal and the β-barrel of these are strikingly different. Our previous results have indicated that the β-barrel may underlie the structural foundation for the anion-selective property of *Tt*MscS (Zhang et al., 2012), but the mechanistic details by which the β-barrel determines anion selectivity are still vague. Therefore, we further analyzed the crystal structure of *Tt*MscS and found three distinct residues—T272, V274, and L276—located in the inner wall of the β-barrel pore, and the V274 residue strikingly pointed at the center of the β-barrel (Figures 3C,D). On the basis of this structural information, we designed a series of mutations and tested their anion preferences by using a giant liposome patch-clamp system.

### Residue V274 plays a crucial role in the ion selection of *Tt*MscS

**Figure 4:**
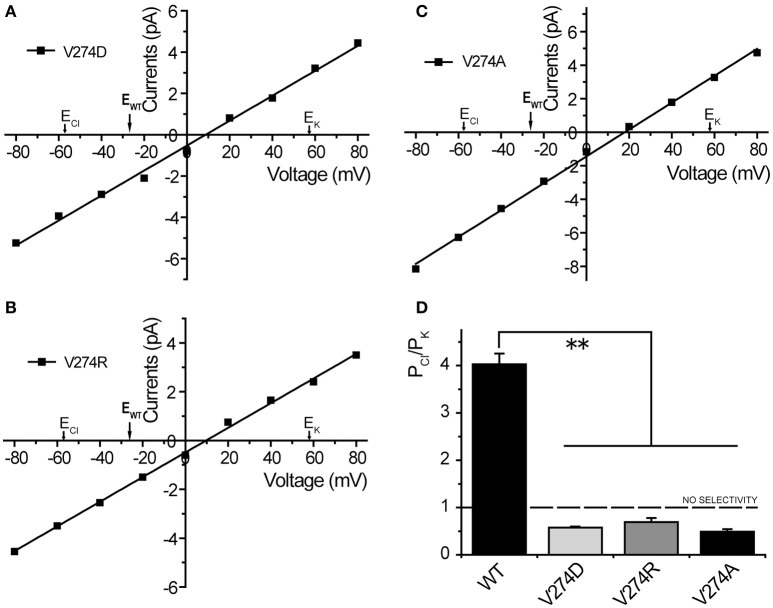
Residue V274 inside the β-barrel plays a vital role in the anion selectivity of *Tt*MscS. **(A–C)** I–V curves for mutations V274D, V274R, and V274A. The reversal potentials were +11.3 ± 1.1 mV (mean ± SEM, *n* = 4), +7.9 ± 2.9 mV (mean ± SEM, *n* = 3), and +15.4 ± 2.0 mV (mean ± SEM, *n* = 7) for V274D, V274R, and V274A, respectively. The arrow (E_WT_) represents the reversal potential position of wild-type *Tt*MscS. **(D)** Summary of ion selectivity (P_Cl_/P_K_) for wild-type *Tt*MscS (WT), V274D, V274R, and V274A mutations. ^**^p < 0.01.

Considering electrostatic potential, we mutated the V274 residue into a negatively charged aspartic acid (V274D) and tested it by using the patch-clamp system in asymmetric KCl solutions (15 mM KCl in pipette and 150 mM in the bath). Surprisingly, the reversal potential shifted from −26.7 ± 0.9 to +11.3 ± 1.1 mV (mean ± SEM, *n* = 4), thus indicating that the V274D mutation reversed the anion selectivity of *Tt*MscS to slight cation selectivity (Figures 4A,D). However, when we mutated the V274 residue to a positively charged arginine (V274R), the reversal potential did not exhibit a striking change compared with that of the V274D mutant (Figures 4B,D, shifted to +7.9 ± 2.9 mV, mean ± SEM, *n* = 3). We mutated V274 to alanine, which has a smaller side chain (V274A). The V274A mutation shifted the reversal potential to +15.4 ± 2.0 mV (Figures 4C,D, mean ± SEM, *n* = 7). Together, the three mutations at V274 neutralized the anion selectivity of *Tt*MscS [Figure 4D, Table 2, one-way ANOVA, *F*_(3, 18)_ = 135.801, *p* < 0.001] thus indicating that the V274 residue plays an essential role in the anion selectivity of *Tt*MscS.

**Table 2:** Summary of properties of wild-type *Tt*MscS (WT) and mutants.

	**E_rev_ (mV)**	**P_Cl_/P_K_**	**Conductance (γ, pS)**	***n***
WT	−26.7 ± 0.9	4.0 ± 0.2	56.8 ± 6.6	7
V274D	+11.3 ± 1.1	0.6 ± 0.1	64.8 ± 9.2	4
V274R	+7.9 ± 2.9	0.7 ± 0.1	77.3 ± 28.2	3
V274A	+15.4 ± 2.0	0.5 ± 0.1	75.4 ± 19.5	7
T272A	−23.7 ± 0.7	3.5 ± 0.1	53 ± 8.2	3
T272K	−9.9 ± 1.4	1.6 ± 0.1	69.8 ± 5.4	4
L276A	−7.5 ± 1.0	1.5 ± 0.1	72.5 ± 14.1	4
L276K	−25 ± 1.2	3.7 ± 0.3	54.7 ± 14.2	3

**Figure 5:**
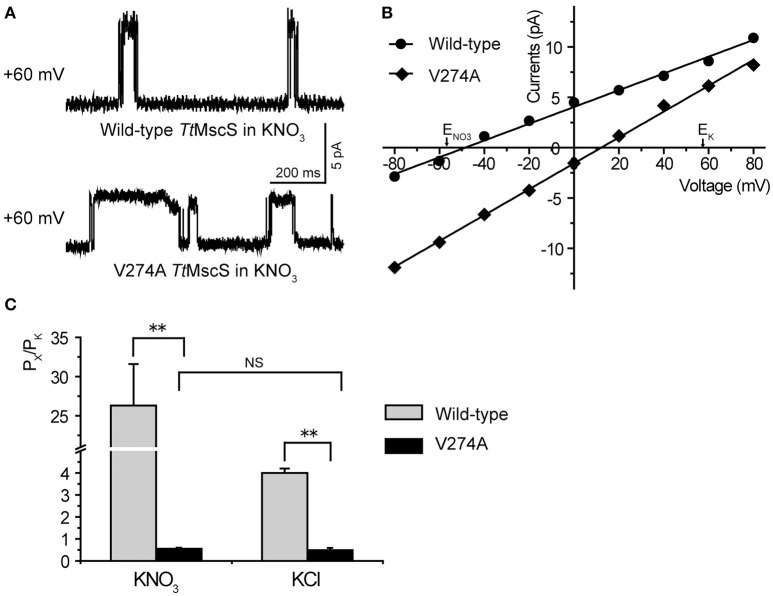
V274 residue is critical for the ${\text{NO}}_{3}^{-}$ selectivity of *Tt*MscS channel. **(A)** Single-channel traces of wild-type *Tt*MscS and V274A mutation in KNO_3_ solution at +60 mV. **(B)** I–V curves for *Tt*MscS and V274A mutation recorded at asymmetric KNO_3_ solution. The reversal potentials of V274A mutation was +11.4 ± 1.6 mV (mean ± SEM, *n* = 5). **(C)** The summary of ion selectivity (P_X_/P_K_: P_NO3_/P_K_ or P_Cl_/P_K_) for wild-type *Tt*MscS and V274A mutation in KNO_3_ and KCl solutions. NS: no significant difference. ^**^p < 0.01.

As shown in Figures 2B,C, wild-type *Tt*MscS displayed much stronger selectivity for ${\text{NO}}_{3}^{-}$ than for the other monovalent anions (Cl^−^, Br^−^, and F^−^). To determine whether V274 residue is also critical for the ${\text{NO}}_{3}^{-}$ selectivity of *Tt*MscS channel, we further tested the reversal potential changes of V274A mutation in asymmetric KNO_3_ solutions. Similar to the situation in KCl solution, V274A reversed the reversal potential from −48.6 ± 1.9 mV (wild type) to +11.4 ± 1.6 (mean ± SEM, *n* = 5; V274A), which indicated that V274A mutation also neutralized the ${\text{NO}}_{3}^{-}$ selectivity of *Tt*MscS channel (Figures 5A–C, Table 3).

**Table 3:** Property comparison of wild-type *Tt*MscS (WT) and V274A mutant in KNO_3_ and KCl solutions.

	**WT**			**V274A mutant**		
	**E_rev_ (mV)**	**P_X_/P_K_**	**Conductance (γ, pS)**	**E_rev_ (mV)**	**P_X_/P_K_**	**Conductance (γ, pS)**
KCl	−26.7 ± 0.9	4.0 ± 0.2	56.8 ± 6.6	+15.4 ± 2.0	0.5 ± 0.1	75.4 ± 19.5
KNO_3_	−48.6 ± 1.9	26.3 ± 5.3	82.3 ± 7.1	+11.4 ± 1.6	0.55 ± 0.1	128.3 ± 22.9
				(*n* = 5)	(*n* = 5)	(*n* = 5)

Collectivity, those results strongly support that V274 residue plays a vital role in the process of monovalent anions selectivity, including selectivity of Cl^−^, ${\text{NO}}_{3}^{-}$, and maybe as well as Br^−^, F^−^, in *Tt*MscS channel.

### The electrostatic potential of residue T272 and the physical size of residue L276 affect the anion selectivity of *Tt*MscS

**Figure 6:**
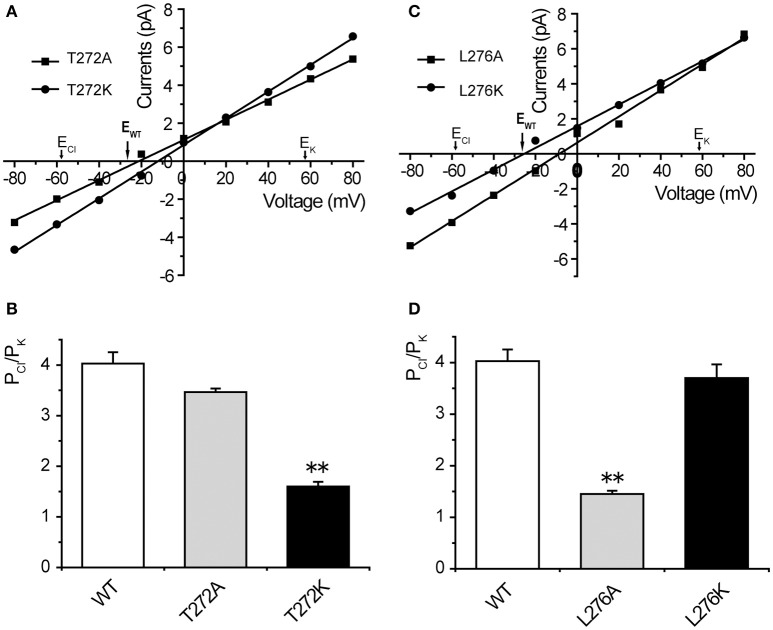
T272 and L276 residues co-determined the anion selectivity of *Tt*MscS. **(A)** I–V curves for T272A and T272K. The reversal potentials were −23.7 ± 0.7 mV (mean ± SEM, *n* = 3) and −9.9 ± 1.4 mV (mean ± SEM, *n* = 4) for T272A and T272K, respectively. The arrow (E_WT_) indicates the reversal potential position of wild-type *Tt*MscS. **(B)** Summary of ion selectivity (P_Cl_/P_K_) for wild-type *Tt*MscS (WT), T272A, and T272K mutations. **(C)** I–V curves for L276A and L276K. The reversal potentials were −7.5 ± 1.0 mV (mean ± SEM, *n* = 4) and −25.0 ± 1.2 mV (mean ± SEM, *n* = 3) for L276A and L276K, respectively. The arrow (E_WT_) indicates the reversal potential of wild-type *Tt*MscS. **(D)** Summary of ion selectivity (P_Cl_/P_K_) for wild-type *Tt*MscS (WT), L276A and L276K mutations. ^**^p < 0.01.

Our structural analysis showed that T272 and L276 are two additional residues located in the inner wall of the β-barrel. To identify the roles of these two residues in the anion selectivity of *Tt*MscS, we mutated T272 and L276 to alanine (small physical size, T272A and L276A) or lysine (positively charged, T272K and L276K), respectively. The electrophysiological results showed that the reversal potential of the T272A mutant, compared with that of wild-type *Tt*MscS, did not exhibit substantial changes (Figures 6A,B, Table 2, E_rev_ = −23.7 ± 0.7 mV, mean ± SEM, *n* = 3). However, the reversal potential of the T272K mutant shifted to −9.9 ± 1.4 mv (mean ± SEM, *n* = 4), thus indicating a significant attenuation of anion selection [Figures 6A,B, Table 2, one-way ANOVA, *F*_(2, 11)_ = 37.443, *p* < 0.001 for Figure 6C]. In addition, the results for the L276 mutant were opposite from those of the V274 mutant. L276A rather than L276K markedly changed the reversal potential [Figures 6C,D, Table 2, for L276A, E_rev_ = −7.5 ± 1.0 mV, mean ± SEM, *n* = 4; for L276K, E_rev_ = −25.0 ± 1.2 mV, mean ± SEM, *n* = 3, one-way ANOVA, *F*_(2, 11)_ = 37.881, *p* < 0.001 for Figure 6D]. From the above comparison, we concluded that the electrostatic potential of T272 and the physical size of L276 to notably influence the anion selection of *Tt*MscS.

## Discussion

Ion selectivity is a basic property of ion channels and is crucial for their physiological function (Roux et al., 2011). In the present study, a single mutation and patch-clamp recording system were used to investigate the mechanism of anion selectivity of *Tt*MscS. Our results suggested that *Tt*MscS is a mechano-sensitive and anion-selective channel. The preference ratio was P_Cl_/P_K_ ~ 4:1, and the free energy of the channel activation was ΔG_o_ = 17.5 ± 2.9 kT. Second, *Tt*MscS showed varying preferences for different anions (the degree order was ${\text{NO}}_{3}^{-}$ > Cl^−^ ≈ F^−^ > Br^−^), especially for ${\text{NO}}_{3}^{-}$, for which *Tt*MscS exhibited a more marked preference as compared with other anions (E_rev_ = −48.6 ± 1.9, P_NO3_/P_K_ ~ 26:1). A conductance comparison suggested that *Tt*MscS had much lower conductance to F^−^ than to other anions (Cl^−^, Br^−^, ${\text{NO}}_{3}^{-}$) larger than F^−^. Third, mutations at the center of the β-barrel (V274) reversed the reversal potentials and thus reversed the ion preference (from anion preference to cation preference) of *Tt*MscS. Finally, changes in electrostatic potential (T272K) and physical size (L276A) of residues in the inner wall of β-barrel both attenuated the anion preference of *Tt*MscS. Together, our results indicated that all central pointed residues located in the inner wall of the β-barrel play a crucial role in the anion selectivity of *Tt*MscS. Generally, functional properties are based on structural domains. The present results suggested that the β-barrel of *Tt*MscS acts as a “selective filter” that confers *Tt*MscS its anion selection.

Mechanisms underlying the anion selectivity of ion channels have been reported previously. However, the location of selective filters varies substantially among anion selective channels. ClC chloride channels belong to a large Cl^−^ channel family found in organisms including bacteria and animals (Dutzler, 2006). In addition to Cl^−^, ClC chloride channels are selective for other small monovalent anions (Br^−^, I^−^, ${\text{NO}}_{3}^{-}$, and SCN^−^; Miller, 2006). Structural studies have indicated that an appropriate hourglass-shaped ion pathway and a partial positive binding site together determine the anion selectivity of ClC channels (Dutzler et al., 2002, 2003; Dutzler, 2007). This finding might describe principles of anion selectivity for anion-selective ion channels, despite differences between ion channels and pumps. In mammals, GABA receptors are ligand-gated chloride channels, which are inhibitory neurotransmitter receptors critical for maintaining appropriate neuronal activity and synaptic transmission (Wotring et al., 2003; Sigel and Steinmann, 2012). However, the mechanism underlying the chloride selectivity of GABA receptors is unclear. Glutamate-gated chloride channel α (GluCl) is a glutamate-gated chloride selective channel that belongs to the same family of GABA receptors (Cys-loop family). A structural analysis has demonstrated that the electropositive concave pocket at the bottom of GluCl channel is crucial for the anion selective property (Hibbs and Gouaux, 2011).

Unlike voltage-gated K^+^, Na^+^, Ca^2+^, and Cl^−^ channels, whose selectivity arises from specific residues in the TM domain, the selectivity of MscS channels is defined by the physical size and charge distribution within the cytoplasmic vestibular domain (Gamini et al., 2011). Quantitative studies have reported that the cytoplasmic equatorial portals act as selective-filters and gateways for ion permeation in MscS channels (Perozo and Rees, 2003; Martinac et al., 2008; Gamini et al., 2011; Cox et al., 2013). However, the structural comparison showed that *Tt*MscS has smaller portals but a larger β-barrel than *Ec*MscS (Zhang et al., 2012). In addition, the size and charge distribution of the cylindrical β-barrel pore in *Tt*MscS is highly similar to the bottom electropositive concave pocket of the GluCl channel (Hibbs and Gouaux, 2011; Zhang et al., 2012). Moreover, the β-barrel is a common structural motif in outer membrane proteins (OMPs) which have been described in the outer membranes of bacteria (Schulz, 2002) and mitochondria (Heins et al., 1994; Hoogenboom et al., 2007). Voltage-gated anion channels (VDAC), which are outer membrane proteins of mitochondria characterized by a transmembrane β-barrel structure, show a slight anion selectivity (Heins et al., 1994). All of these findings suggest that the β-barrel of *Tt*MscS might reasonably be capable of ion permeation and ion selection. In the present study, a single residue mutation at the center of the β-barrel (V274) neutralized and even reversed the anion selectivity of *Tt*MscS, thus suggesting a crucial role for residue locates at the center of β-barrel for the anion selectivity of *Tt*MscS. Mutations of the other two extrusive residues at the inner wall of the β-barrel both attenuated anion selectivity, thus further verifying the crucial role of the β-barrel in the anion selectivity. The presented results suggest that the β-barrel acts as a “selective filter” module at the distal of anion selection of *Tt*MscS. Complex proteins comprise a series of functional domains that endow the characteristic properties of *Tt*MscS and confers this channel with a moderate degree of anion selectivity.

The MS channel responds to mechanical tension along the plane of the membrane. Studies have shown that the movement of transmembrane helices plays a critical role in the gating process of MscS channels (Martinac, 2004; Sotomayor and Schulten, 2004), suggesting that TM1 and TM2 may be responsible for gating the channel (Böttcher et al., 2015). In addition, the interaction of charged and polar residues in the TM1-TM2 helices with lipid headgroups can also affect the gating of MS channels (Sotomayor and Schulten, 2004). In the present study, the results showed that the *Tt*MscS channel was open under negative pressure, but the negative pressure corresponding to the 1/2 channel open probability (P_1/2_) of *Tt*MscS was much larger than that for *Ec*MscS (−79.7 vs. −36 mmHg), even though both proteins belong to the same family and have very similar structures. The different pressure sensibilities between *Tt*mscS and *Ec*MscS may arise from the distinct arrangement of amino acid residues in the TM domain.

In summary, our results provide new insight into the molecular mechanism of the ion permeability and selectivity of the *Tt*MscS channel. Our results indicate that both the electrostatic potential and the physical size of side chains inside the “selective filter” determine the anion selectivity of *Tt*MscS, and thus may aid in understanding the mechanism of substrate selectivity of OMPs and the anion selectivity of chloride channels in eukaryotes.

## Author contributions

YS, BZ, and FG: Performed all the experiments. BZ, MY, and YL: Initiated the project. All the authors contributed to data analysis and paper writing.

### Conflict of interest statement

The authors declare that the research was conducted in the absence of any commercial or financial relationships that could be construed as a potential conflict of interest.
